# Pindex: Private multi-linked index for encrypted document retrieval

**DOI:** 10.1371/journal.pone.0256223

**Published:** 2021-08-20

**Authors:** A. John Prakash, B. Lydia Elizabeth

**Affiliations:** 1 Ramanujan Computing Centre, Anna University, Chennai, TN, India; 2 Department of IT, Anna University, Chennai, TN, India; Sri Eshwar College of Engineering, INDIA

## Abstract

Cryptographic cloud storage is used to make optimal use of the cloud storage infrastructure to outsource sensitive and mission-critical data. The continuous growth of encrypted data outsourced to cloud storage requires continuous updating. Attacks like file-injection are reported to compromise confidentiality of the user as a consequence of information leakage during update. It is required that dynamic schemes provide forward privacy guarantees. Updates should not leak information to the untrusted server regarding the previously issued queries. Therefore, the challenge is to design an efficient searchable encryption scheme with dynamic updates and forward privacy guarantees. In this paper, a novel private multi-linked dynamic index for encrypted document retrieval namely Pindex is proposed. The multi-linked dynamic index is constructed using probabilistic homomorphic encryption mechanism and secret orthogonal vectors. Full security proofs for correctness and forward privacy in the random oracle model is provided. Experiments on real world Enron dataset demonstrates that our construction is practical and efficient. The security and performance analysis of Pindex shows that the dynamic multi-linked index guarantees forward privacy without significant loss of efficiency.

## 1 Introduction

Cloud computing has revolutionized data storage by offering effortless data storage for personal, enterprises, governments and institutions. Data owners have flexible, on-demand access to data with simplified data management and significant cost benefits with increased performance. Data owners outsource sensitive data such as the Electronic Health Records (EHR), citizens’ personal information, emails, credit cards data, government information and critical business data to the public cloud [1]. Despite the numerous benefits of cloud storage fuelled by high speed networking technologies and although the cloud storage providers (CSPs) claim to adopt strong security measures governments, organizations and businesses are slow to fully embrace the public cloud storage due to privacy and security concerns. When data owners outsource sensitive data to the cloud; they lose control over their data leading to a number of security issues [2].

Data owners encrypt their sensitive information to protect privacy of data stored with the honest but curious cloud storage and to defend against unauthorized access. Encryption imposes restriction on searching which is the only way to access the data. Unless they can easily be indexed, shared, retrieved and utilized, encrypted data serves no purpose. Searchable encryption enables a user to perform efficient keyword search while preserving privacy. Indexes are used to improve the search performance by significantly reducing the search complexity. A number of searchable encryption schemes [3–6] have been proposed but most of the schemes provide a static mechanism which hinders its application in reality.

Dynamic searchable encryption allows clients to dynamically update files or keywords without rebuilding the encrypted index. For any searchable encryption scheme to be practical the scheme must be secure and should allow efficient updates with optimal search time. A few reported works [7–11] support dynamic index. Nevertheless, most of the solutions leaks critical information since the adversary can observe and learn more information from the interaction between data user and CSP. This leakage can be from search pattern, access pattern, update pattern, size pattern, file identifiers containing a specific keyword, trace and trapdoor linkability. As a result of the leakages, forward privacy was introduced by Stefanov et al. [12]. Zhang et al. [13] presented a file injection attack on dynamic constructions by adding files to the database. There is a trade-off between security and practicality. In addition to the data privacy, it is imperative that the computational correctness should be guaranteed by design. This is because the malicious server can return incorrect results due to hardware or software malfunction or to save resources. In order to fully utilize the services offered by the cloud server, there is a need for a provably secure dynamic searchable encryption scheme with efficient search, forward privacy and support for parallelism, which is a challenging problem.

From the above discussions, the significant research problems in designing protocols for privacy-preserving search over encrypted outsourced cloud data can be summarized as: a) an efficient and secure index construction to improve search without reconstructing the index. b) a privacy preserving search over encrypted data with allowable minimum leakage of information to the CSP. c) providing support for efficient dynamic updates to encrypted index with allowable minimum leakage and without requirement of trusted platform at the CSP. d) Support parallel execution of multi-keyword search and update operations. e) a construction with minimum communicational and computational overhead. Motivated by the challenges summarized above the main contributions and results of the proposed method, namely, Pindex are:

1. *Secure Dynamic Index*: A novel private multi-linked dynamic index construction using probabilistic homomorphic encryption and a secret orthogonal vector as building blocks. Support to add/delete keywords or documents without reconstructing the outsourced encrypted index using the hash table that contains the sum of inner product of the rows that are linked by orthogonal vectors.
2. *Forward Privacy*: The key used in construction of the index and trapdoor or token generation are indistinguishable from random functions. This is because Pindex uses probabilistic homomorphic encryption and secret orthogonal vectors. Thus, the server cannot learn information from search or access patterns from previous queries.
3. *Efficient and Parallelizable*: The server performs $O(|F_{q}|)$ and $O(|W_{f}|)$ functions evaluations for each search and update operation where |*F*_*q*_| is the number of documents that contain the query keyword *w*_*q*_ and |*W*_*f*_| is the number of keywords contained in a file *f* to be updated. The client and server storage overhead is reduced to O(n). The multi-linked index structure allows multi-keywords search and update operations to run independently over *p* processors.
4. The results obtained from security analysis shows that the proposed design is secure in the real-ideal security model, efficient with sub-linear time complexity $O(|F_{q}|)$, efficiently parallelized and exhibits relatively better performance in empirical analysis.

The rest of the paper is organized in the order of research methodology adopted which is similar to the standard works [5, 8, 9, 12, 14, 15]. First, we define the security requirements of the protocol under design in the form of definitions such that it captures thoroughly the adversarial environment and adversary. Such definitions stated as a security model for the protocol to serve as the basis for further design is described in section 2. Second, we describe in section 3 the design of the cryptographic protocol adhering to the security model devised in section 2. Third, we present in section 4, a thorough theoretical correctness and security analysis using standard provable security attack or adversary model to investigate vulnerabilities in the design. Fourth in section 5, we support the theoretical correctness and efficiency with results obtained from empirical simulations. Lastly, we present in section 6 the related works, followed by discussion in section 7 and conclusion in section 8.

## 2 Model

This section describes the preliminaries and security model which captures the requirements of the protocol under design. The model captures the notions of security and adversary through precise definitions to be used later as the basis of design.

### 2.1 Notations and system model

**Fig 1:**
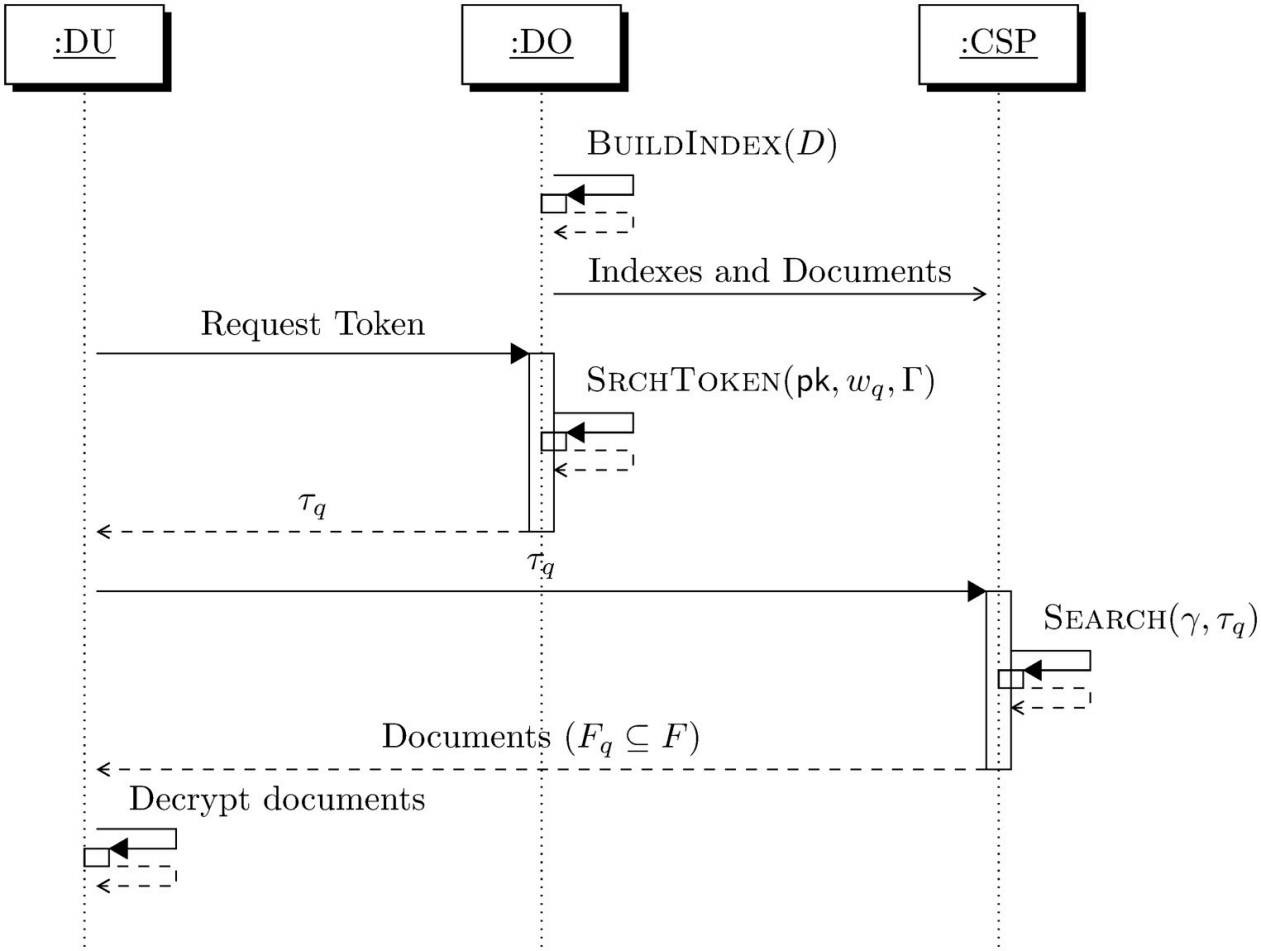
System model.

Let *D* = {*d*_1_, … *d*_*n*_} be the set of document collection, *C* = {*C*_1_, … *C*_*n*_} be the encrypted collection of documents and *W* = {*w*_1_, …, *w*_*m*_} be the set of keywords. Let *F* = {*f*_1_, …, *f*_*n*_} denote the file identifiers of documents given by *f*_*i*_ = *id*(*C*_*i*_). A collection of document *D* is to be outsourced to the Cloud Service Provider (CSP). The data owner encrypts the document set *D* to obtain a encrypted document collection *C*. To enable privacy preserving searching over the encrypted document collection *C*, a secure encrypted index denoted by *γ* is built using BuildIndex. The encrypted document collection *C*, the secure encrypted Index *γ* are outsourced to the CSP. An authorised Data User (DU) acquires a search token denoted by *τ*_*q*_ generated using SrchToken corresponding to a given query keyword *w*_*q*_ from the Data Owner (DO). The search token *τ*_*q*_ is then sent to the CSP using a standard communication protocol. The CSP searches for the matching documents for the query keyword using Search over the encrypted index *γ* preserving privacy. The Search algorithm outputs a set of file identifiers relevant to the query denoted by *F*_*q*_ that contain the query keyword. The DO updates the index using Update whenever the document changes or files need to be added/deleted. The overall system working mechanism of the search is shown in Fig 1. We use the notation = to denote a deterministic assignment, ← a probabilistically computed assignment and ←_$_ an uniformly sampled assignment.

### 2.2 Security definitions

The construction of the Pindex scheme aims to securely perform computations at the CSP on the encrypted data while preserving privacy and is defined as in [9]:

**Definition 1** (Pindex). Pindex *is a dynamic searchable encryption scheme consisting of a tuple* {KGen, Enc_1_, Dec_1_, Enc_2_, Dec_2_, BuildIndex, SrchToken, Search, UpdToken, Update} *of ten algorithms such that*:

1. *K* ← KGen(1^λ^) *is a probabilistic polynomial time algorithm that outputs a secret key K* ← {pk, sk, *k*} *when given as input a security parameter* 1^λ^
2. *c* ← Enc_1_(*k*, *f*) *is a CPA-secure deterministic polynomial time algorithm that outputs a sequence of encrypted ciphertexts c when given as input a secret key k and a file f*.
3. *f* = Dec_1_(*k*, *c*) *is a deterministic polynomial time algorithm that outputs a file f when given as input a secret key k and a ciphertext c*.
4. *c* ← Enc_2_(pk, *m*, *r*) *is a CPA-secure probabilistic polynomial time additive homomorphic algorithm that outputs an encrypted ciphertext c when given as input a secret key* pk, *a message m and randomness r*.
5. *m* = Dec_2_(sk, *c*) *is a deterministic polynomial time algorithm that outputs m when given as input a secret key* sk *and a ciphertext c*.
6. (*γ*, Γ) ← BuildIndex(*K*, *δ*, *V*, *D*, Enc_2_) *is a probabilistic polynomial time algorithm that outputs a encrypted index γ and a parameter hash table* Γ *when given as input a secret K, vector V, primitive* Enc_2_, *document collection D and a index δ*.
7. *τ_q_* ← SrchToken(pk, *w_q_*, Γ) *is a probabilistic polynomial time algorithm that outputs a search token τ_q_ when given as input a secret key* pk, *query keyword w_q_ and a parameter hash table* Γ.
8. *F_q_* ← Search(*γ*, *τ_q_*) *is a deterministic polynomial time algorithm that outputs a set of identifiers F_q_ ⊆ D when given as input an encrypted index γ and a search token τ*_*q*_.
9. *τ_u_* ← UpdToken(pk, *f_i_*, *u*, *w_u_*) *is a probabilistic polynomial time algorithm. Given a secret key K, update keyword w_u_ and a file f_i_ as input, the algorithm outputs an update token τ_u_ for the update type u* ∈ {*add*, *delete*}.
10. *γ*′ ← Update(*γ, τ_u_*) *is a deterministic polynomial time algorithm that outputs an updated encrypted index γ′ given an encrypted index γ and an update token τ*_*u*_.

A scheme as defined above is secure when it is designed based on the real/ideal security paradigm. The paradigm requires defining correctness and privacy. The correctness captures the notion that the specified polynomial time algorithms in Definition 1 outputs correctly and it is given as:

**Definition 2 (Correctness)**. *Let π be a dynamic searchable encryption scheme as given in Definition 1. Such π is said to be correct if*∀k∈N, ∀*K*: *K* ← KGen(1^λ^), ∀(*δ*, *f*), ∀*γ*: *γ* ← BuildIndex(*K, δ, V, D*, Enc_2_), ∀*c*: *c* ← Enc_1_(*k*, *f*) *and for all update operations of* Update(*γ, τ_u_*), where *τ*_*u*_ *is the update token obtained by* ∀(*f, τ_u_*): *τ_u_* ← UpdToken(pk, *f_i_, w*) *and all u* ∈ {*add*, *delete*}, ∀(*w, τ_q_*: *τ_q_* ← SrchToken(pk, *w*, Γ), ∀*F_q_*: *F_q_* ← Search(*γ, τ_q_*), *the plaintext f*_*w*_ = {Dec_1_(*k*, *c*_*i*_):*i* ∈ *F*_*q*_} *are all plaintexts in f containing keyword w*.

Most of the dynamic searchable encryption schemes leak information which could be exploited to launch attacks such as file injection attacks to recover the encryption key. For example, information leak such as file identifiers returned for a respective search or update query cannot be prevented. The leakage function captures the notion of allowable leakages in a dynamic searchable encryption defined as in [9]:

**Definition 3 (Leakage)**. *The leakage functions are defined as follows*:

1. *Search Pattern*P(δ,q,t): *It is defined by a one dimensional binary matrix of length m* × *n*. *Given a search keyword w at t, if the search at time i* ≤ *t is for the keyword w then it is marked as 1 at i and 0 otherwise*.
2. *Access Pattern* Δ(*δ*, *f*, *w*, *t*): *It is defined as the set of identifiers f_w_ at time t given a search query for keyword w at time t*.
3. $L_{1}\left(\delta ,f\right)$: *The function outputs the number of keywords m, the number of files n, the identifiers i of the file and the size of each file when given the index δ and set of file identifiers*.
4. $L_{2}\left(\delta ,f,w,t\right)$: *The function outputs*P(δ,q,t)*and* Δ(*δ*, *f*, *w*, *t*) *for a search query at time t*, *given the index δ, set of files f and keyword w as input*.

The definition of privacy or security captures the notion that the view of each party can be separately simulated given the leakage function and it is defined as:

**Definition 4 (Security)**. *Let π be a dynamic searchable encryption scheme as defined in Definition 1. Let A be a stateful adversary, Sim be a stateful simulator and*$\left(L_{1},L_{2}\right)$*are stateful leakage algorithms in the following experiments*:

1. ${\text{Real}}_{A}\left(\lambda \right)$: *The challenger*C*runs K* ← KGen. *The adversary outputs* (δ,f)←A *and receives* (*γ*, Γ) ← BuildIndex(*K, δ, V, D*, Enc_1_) *from* C. *Each time the adversary computes q* ∈ {*w*, *f*_*i*_} *and makes polynomial number of adaptive queries*. A *obtains from* C *a search token τ_q_* ← SrchToken(pk, *w*, Γ) *whenever it wants to perform a search query q* = *w*. *If* A *wants to make a update query q* = *f*_*i*_ *it receives a update token τ*_u_ ← UpdToken(pk, *f_i_*, *w_u_*) *from* C. A *returns an output bit b at the end of the experiment*.
2. ${\text{Ideal}}_{A,S}\left(\lambda \right)$: *Sim outputs* (*γ*, *c*) *to* A *when given* (*δ*, *f*) and $L_{1}\left(\delta ,f\right)$. A *then can make polynomial number of adaptive queries q* ∈ {*w*, *f*_*i*_}. A *makes a search query q* = *w then the simulator is given* $L_{2}\left(\delta ,f,w,t\right)$ *and when q is update query, the Sim is given a new* $L_{2}\left(\delta ,f,w,t\right)$ *including the adaptive query history by* A. *Also, the Sim returns a token τ to* A *appropriately*. A *then returns an output bit b at the end of the experiment*.

*The scheme π is said to be*$\left(L_{1},L_{2}\right)$–*secure against adaptive dynamic chosen-keyword attacks if for all* PPT *adversaries A, there exists a* PPT *simulator* Sim *such that*

$$\begin{array}{c} \text{Pr}[{\text{Real}}_{A}\left(k\right)=1]-\text{Pr}[{\text{Ideal}}_{A,\text{Sim}}\left(k\right)=1]\leq \text{negl}(\lambda ) \end{array}$$ (1)

A dynamic searchable scheme is considered secure if no information is revealed during execution of all its operations. However, designing such schemes which do not leak any information is a challenge. For example, when a search or update is performed for the keyword *w*, the CSP can correlate the file identifiers with it’s query and learn about the outsourced data. Therefore, there is a need for stronger security guarantees for dynamic searchable schemes.

**Definition 5** (Forward private). *A dynamic searchable encryption scheme which is* $\left(L_{1},L_{2}\right)$-*adaptively secure is said to be forward private if the following holds*:

$$\begin{array}{c} L^{upd}\leftarrow L^{\prime }\left(op,\left\{f_{i},\mu_{i}\right\}\right) \end{array}$$ (2)

*where* $L^{upd}$ *is the update leakage function*, {*f*_*i*_, *μ*_*i*_} *is the set of document-number of keywords updated pair*, *f*_*i*_ *is the document updated*, *μ*_*i*_ *is the number of keywords updated on respective f*_*i*_ *and* $L^{\prime }$ *a stateless function*.

The forward private definition 5 states that the server should be able to learn only about the file identifier(s), size of file(s) and number of keywords contained in file(s) after observing searches before and after a dynamic update operation *op* ∈ {Add, Delete} over encrypted index is performed.

### 2.3 Homomorphic and orthogonality

The design of dynamic searchable schemes satisfying definitions 4 and 5 more often requires the use of encryption mechanisms called homomorphic cryptosystems as a building block.

**Definition 6** (Homomorphic). *A public key encryption scheme E* = (KGen, Enc, Dec, *M*, *C*) *is said to be homomorphic if it holds that* Dec(sk, *c*_1_ ⋄ *c*_2_) = *m*_1_ ⋄ *m*_2_ *for all m*_1_, *m*_2_ ∈ *M and c*_1_, *c*_2_ ∈ *C with m*_1_ = Dec(sk, *c*_1_) *and m*_2_ = Dec(sk, *c*_2_) *such that for all c obtained from* Enc(pk, *m*), *c belongs to C*.

The proposed Pindex uses Paillier cryptosystem to instantiate the encryption which exhibits homomorphic addition property. The group operation ⋄ for Paillier cryptosystem has homomorphic property such that Dec(sk, *c*_1_ ⋄ *c*_2_) = *m*_1_ + *m*_2_ [16]. Let *V* = *v*_1_, *v*_2_ …, *v*_|*W*|_ be a matrix of mutually orthogonal vectors where *v*_*i*_ is the *i*^*th*^ row vector and |*W*| is the number of keywords in the keyword collection *W*. A square *n* × *n* matrix *V* with elements ±1 that satisfies *V* × *V*^*T*^ = *nI*_*n*_ is called a Hadamard matrix of order *n* [17, 18]. Such matrices make cross correlation values to be zero and this property is used in the design of the proposed search and update mechanism of Pindex.

## 3 Construction

The cloud data storage service involves three entities namely, the Data Owner (DO), Cloud Server or the Cloud Service Provider (CSP) and the Data User (DU). The index is constructed using a novel multi-linked hash map structure. The index construction based on the security model presented in section 2 is given below:

### 3.1 Multi-linked hash map construction

**Fig 2:**
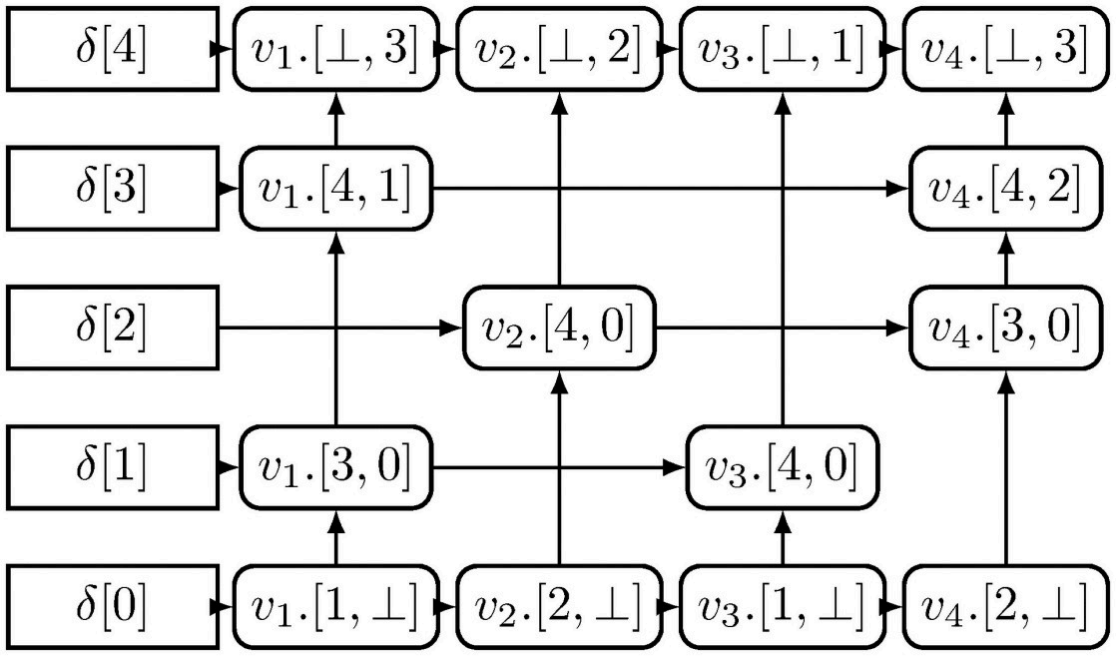
Multi-linked hash map *δ*.

The two dimensional matrix *M*_*mn*_ whose elements are stored in a multi-linked hash map *δ*, where each element {*m*_*ij*_: *m*_*ij*_ = [*next*, *prev*], *m*_*ij*_ ∈ *M*_*mn*_,1 ≤ *i* ≤ *m*,1 ≤ *j* ≤ *n*} has link to the next non-empty element in the respective row *i* and column *j*. The row links are implemented by orthogonal vectors and respective column links using pointers as shown in Fig 2. The hash table value *δ*(*i*) is orthogonally linked row values for all 1 ≤ *i* ≤ *m* as given below:

$$\begin{array}{c} \delta \left(i\right)=\{\sum_{j=1}^{n}v_{j}.m_{ij}:m_{ij}\neq \emptyset \} \end{array}$$ (3)

Eq 3 shows that each value is a sum of inner product of the orthogonal vector *v*_*j*_ and a pointer to the next non-empty value in the column *j*. Each *v*_*j*_ represents a column and all *v*_*j*_’s are mutually orthogonal 4.

$$\begin{array}{c} \left\langle v_{a},v_{b}\right\rangle =\{\begin{array}{cc} 1, & \text{if}\;a=b\\ 0, & \text{otherwise} \end{array} \end{array}$$ (4)

Hence, assuming *y* such that *y* = *j*,

$$\begin{array}{cc} \left\langle v_{y},\delta \left(i\right)\right\rangle & =\langle \sum_{j=1}^{n}v_{j}.m_{ij},v_{y}\rangle \\ & =v_{1}.m_{i1}.v_{y}+\cdots +v_{j}.m_{ij}.v_{y}+\cdots +v_{n}.m_{in}.v_{y}=m_{ij} \end{array}$$ (5)

where *m*_*ij*_ by definition is a pointer to the next non-empty element in the column *j* and <.,.> denotes vector inner product. Therefore, all the non-zero elements in the column *j* can be retrieved using the recursive function:

$$\begin{array}{c} x_{i+1}=\{\begin{array}{cc} \left\langle v_{y},\delta \left(x_{i}\right)\right\rangle & \text{if}\;x_{q}\neq ⊥\\ ⊥, & \text{otherwise} \end{array} \end{array}$$ (6)

The hash table value *δ* can be used as an index for retrieving documents containing a given keyword. Let the rows and columns in matrix *M* represent documents and keywords respectively. Each element in a column *j* is a pointer to next document containing the keyword *w*_*j*_. Each row *i* is encoded as an orthogonal sum of product of keyword vector *v*_*j*_ and the next document *f*_*i*_ containing keyword *w*_*j*_ and stored in a keyword multi-linked hash map as *δ*(*f*_*i*_). Each element *m*_*ij*_ = [*f*_*next*_, *f*_*prev*_] represents the next and previous file of *f*_*i*_ containing the keyword *w*_*j*_ and an empty value *m*_*ij*_ = ∅ represents that *w*_*j*_ ∉ *f*_*i*_. Therefore, using Eq 6 set of all documents *F*_*q*_ containing query keyword *w*_*q*_ can be retrieved in *F*_*q*_ ⊆ *F* in sub-linear time as for all practical queries |*F_q_*| ⋘ |*F*|. The multi-linked hash map data structure is designed as described above such that it supports addition of new documents which includes encoding a row, adding encoded value and updating either the last or first row pointers accordingly. The multi-linked hash map data structure design improves search to sub-linear time and supports parallel execution of search and updates.

### 3.2 Algorithm *K* ← KGen(1^λ^)

It is a probabilistic polynomial time algorithm which generates the secret parameters used for building index and encryption. The Pindex algorithm uses Paillier Cryptosystem for building the index and generation of search and update tokens. The key (pk, sk) generation setup is described in [18] where pk denotes the public key and sk denotes the private key of Paillier cryptosystem. The KGen also generates a secret key *k* to be used by Enc_1_ for encrypting the document collection *D*. The design choice for Enc_1_ is the CPA-secure AES while the design choice for Enc_2_ is the CPA-secure Paillier cryptosystem which also exhibits additive homomorphic properties.

### 3.3 Algorithm *c* ← Enc_2_(*K*, *m*, *r*)

It is a probabilistic algorithm, given a message *m* ∈ *P* and a public key pk = (*n*, *g*), Enc_2_(pk, *m*, *r*) returns the ciphertext *c* = *g^m^r^n^* mod *n*^2^, where given *r* is a random value chosen such that $r\in Z_{n}^{*}|gcd\left(r,n\right)=1$.

### 3.4 Algorithm *m* = Dec_2_(sk, *c*)

It is a probabilistic algorithm, given a ciphertext *c* ∈ *C* and a private key sk = (*p*, *q*, λ), Dec_2_(sk, *c*) returns the message *m*.

$$\begin{array}{c} m=\frac{L(c^{\lambda }\;\mathrm{mod}\;n^{2})}{L(g^{\lambda }\;\mathrm{mod}\;n^{2})}\;\mathrm{mod}\;n \end{array}$$ (7)

### 3.5 Algorithm (*γ*, Γ) ← BuildIndex(*K*, *δ*, *V*, *D*, Enc_2_)

BuildIndex algorithm constructed by the data owner takes as input five parameters namely the document set *F* = {*f*_1_, *f*_2_,…, *f*_|*D*|_}, keyword set *W* = {*w*_1_, *w*_2_,…*w*_|*W*|_}, a pre-built multi-linked hash map *δ* as described in section 3.1 for *F*, mutually orthogonal vector *V* and the key *K* as input. The algorithm outputs multi-linked hash map index *γ* and keyword parameter hash table Γ. The algorithm computes:

1. For each row *i* or file *f*_*i*_ in *δ*:
   For each column *j* or word *w*_*j*_ in *f*_*i*_:
    if *m*_*ij*_ ≠ ∅ and Γ(*w*_*j*_) = ∅ then compute Γ(*w*_*j*_) = (*v* ←_$_ *V*_*w*_, *r* ←_$_ *Z*_*n*_)
     Compute *s* ← Enc_2_(pk, *w*_*j*_, *r*)
     Compute *γ*(0) = *γ*(0) + *v*.*s*.*f* and *γ*(*f*_*i*_) = *γ*(*f*_*i*_) + *v*.*s*.⊥
    if *m*_*ij*_ ≠ ∅ and Γ(*w*_*j*_) ≠ ∅ then retrieve (*v*, *r*) = Γ(*w*_*j*_)
     Compute *s* ← Enc_2_(pk, *w*_*j*_, *r*)
     if *f*_*next*_ ≠ ∅ then compute *γ*(*f*_*i*_) = *γ*(*f*_*i*_) + *v*.*s*.*f*_*next*_ otherwise *γ*(*f*_*i*_) = *γ*(*f*_*i*_) + *v*.*s*.⊥
   Compute *v*_*r*_ ←_$_ *V*_*r*_ and *r*′ ←_$_ *Z*_*m*_
   Compute *γ*(*f*_*i*_) = *γ*(*f*_*i*_) + *v*_*r*_.*r*′
2. Return (*γ*, Γ)
3. Send *γ* to CSP and Γ is kept secret at the DO

**Fig 3:**
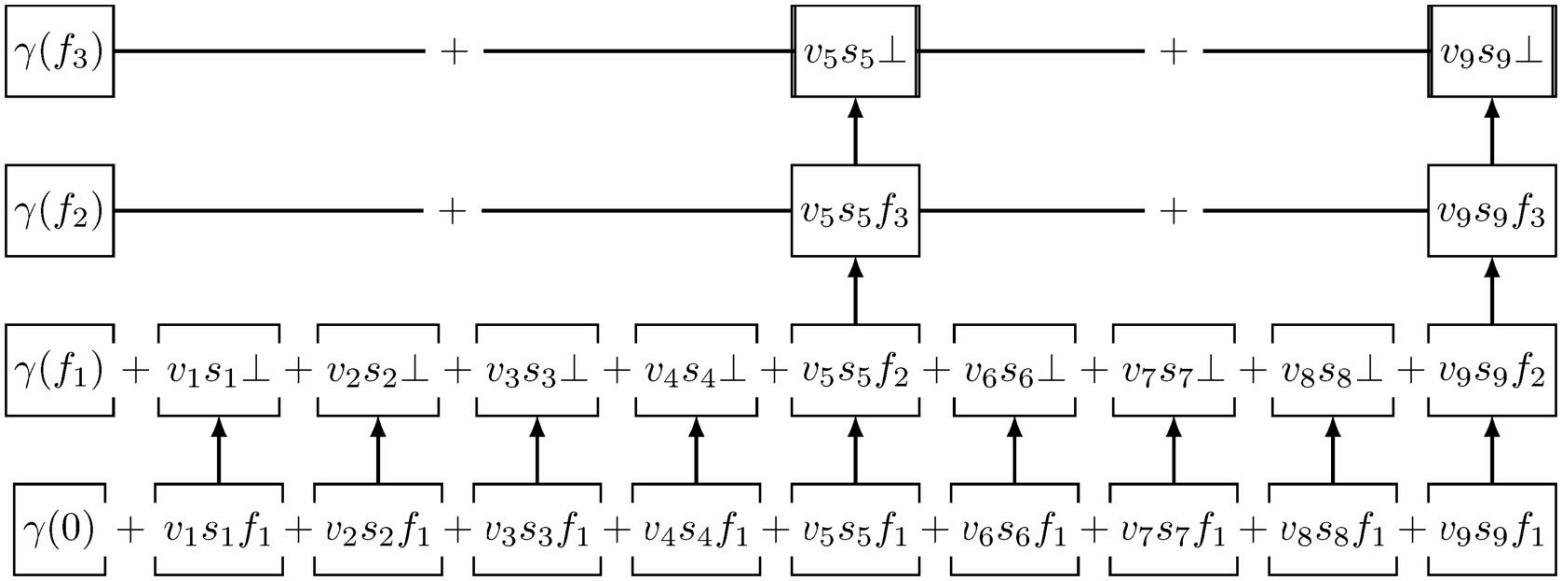
Index structure.

The pictorial representation of index structure is given in Fig 3. The encrypted index (*γ*, Γ) can be observed to be designed such that the link can be traversed vertical through the pointers and traversed horizontal through the orthogonal property without leaking critical information.

### 3.6 Algorithm *τ*_s_ ← SrchToken(*w_q_*, pk, *V*)

The SrchToken algorithm is computed by the Data Owner. It takes a query keyword *w*_*q*_ ∈ *W*, key pk, set of mutually orthogonal vectors *V* as input and outputs a search token *τ*_*q*_. The algorithm is given below:

1. Get the keyword secret (*v*, *r*) ← Γ(*w*_*q*_) from the keyword parameter hash table Γ and compute $r^{-1}\in Z_{n}$ such that *rr*^−1^ ≡ 1 mod *n*^2^
2. Compute *s*^−1^ ← Enc_2_(pk, −*w*_*q*_, *r*^−1^) and returns to DU the search token *τ*_*q*_ by computing:
  $$\begin{array}{c} \tau_{q}\leftarrow v.s^{-1}+v^{\prime }.r^{\prime } \end{array}$$ (8)
  where $r^{\prime }\leftarrow_{\$}Z_{n}$ and *v*′ ←_$_ *V*_*r*_

The trapdoor *τ*_*q*_ is designed to contain the encrypted keyword parameters embedded with the corresponding orthogonal vector *v* and random *v*′.*r*′ are added at each instance to make the token appear indistinguishable even when the same query is repeated.

### 3.7 Algorithm *F_q_* ← Search(*γ, τ_q_*)

The Search algorithm is computed at CSP and it takes as input the encrypted index *γ*, search token *τ*_*q*_, key pk and outputs *F*_*q*_ = {*f*_1_, *f*_2_,‥*f*_*n*_}. *F*_*q*_ is the set of document identifiers such that *w*_*q*_ satisfies *w*_*q*_ ∈ *W* and ⊥ otherwise, where ⊥ denotes null. The search algorithm is given below:

1. Compute 〈*γ*(0), *τ*_*q*_〉 to obtain the first file pointer *f*_*q*_ = *f*_1_ of document containing the keyword *w*_*q*_.
2. If *f*_*q*_ = ∅ then there are no documents matching the query keyword *w*_*q*_ or the search token is invalid. The algorithm terminates returning ⊥.
3. Otherwise, obtain all *f*_*q*_ computing recursively as given below with base condition *f*_*q*_ = *f*_1_ until *f*_*q*_ = ⊥
  $$\begin{array}{c} f_{q+1}=\{\begin{array}{cc} \left\langle \tau_{q},\gamma \left(f_{q}\right)\right\rangle & \text{if}\;f_{q}\neq ⊥\\ ⊥, & \text{otherwise} \end{array} \end{array}$$ (9)
4. Return all file identifiers {*f*_1_, …, *f*_*q*_, …, |*F*_*q*_|} obtained.

Thus, it can be observed that the design of the search algorithm is simple and dependent on the search token design. Since, the search token is designed to be created with the orthogonally embedded encrypted keyword parameters (*v*.*s*^−1^) and the index contains (*v*, *s*), a inner product of the two will result in retrieving the corresponding document identifiers. The proof of correctness for which is presented in section 4.

### 3.8 Algorithm *τ_q_* ← UpdToken(pk, *f*, *u*, *w*)

The UpdToken probabilistic algorithm is computed by the DO. The algorithm returns an update token to the CSP for securely updating the index. We define a header node to be the node pointed by *γ*(0) or the row when *i* = 1 in *δ*, a terminal node to be the last file containing ⊥ for a *w* in *δ* and any other node as intermediate nodes containing a keyword *w*. The algorithm computes:

1. When the update operation is DeleteKeyword *w* ∈ *W* from document *f* then compute:
  - Get (*v*, *r*) ← Γ(*w*), compute *s* ← Enc_2_(pk, *w*, *r*)
  - When *f* is the header node, compute *τ*_1_ ← *v*.*s*.*f*_*next*_, *τ*_2_ ← *v*.*s*.*f* and return *τ* ← (*τ*_1_, *τ*_2_, *f*_1_ = 0, *f*_2_ = *f*)
  - When *f* is the terminal node, compute *τ*_1_ ← *v*.*s*.⊥, *τ*_2_ ← *v*.*s*.*f* and return *τ* ← (*τ*_1_, *τ*_2_, *f*_1_ = *f*_*prev*_, *f*_2_ = *f*)
  - When *f* is an intermediate node, determine *f*_*next*_, *f*_*prev*_, compute *τ*_1_ ← *v*.*s*.*f*_*next*_, *τ*_2_ ← *v*.*s*.*f* and return *τ*_*u*_ ← (*τ*_1_, *τ*_2_, *f*_1_ = *f*_*prev*_, *f*_2_ = *f*)
2. When the update operation is DeleteFile for a document *f* then compute:
  - Get (*v*, *r*) ← Γ(*w*), compute *s* ← Enc_2_(pk, *w*, *r*),
  - When *f* is the header node, compute
    $$\begin{array}{cc} \tau_{1} & =\tau_{1}\cup v_{i}.s_{i}.f_{next},\;1<i<\left|\forall w\in f\right|\\ f_{1} & =f_{1}\cup f_{next,i},\;1<i<\left|\forall w\in f\right|\\ \tau_{2} & =\tau_{2}\cup v_{i}.s_{i}.f,\;1<i<\left|\forall w\in f\right| \end{array}$$
    and return *τ* ← (*τ*_1_, *τ*_2_, *f*_1_, *f*_2_ = *f*)
  - When *f* is the terminal node, compute
    $$\begin{array}{cc} \tau_{1} & =\tau_{1}\cup v_{i}.s_{i}.⊥,\;1<i<\left|\forall w\in f\right|\\ f_{1} & =f_{1}\cup f_{prev,i},\;1<i<\left|\forall w\in f\right|\\ \tau_{2} & =\tau_{2}\cup v_{i}.s_{i}.f,\;1<i<\left|\forall w\in f\right| \end{array}$$
    and return *τ* ← (*τ*_1_, *τ*_2_, *f*_1_, *f*_2_ = *f*)
  - When *f* is an intermediate node, determine *f*_*next*_, *f*_*prev*_, compute
    $$\begin{array}{cc} \tau_{1} & =\tau_{1}\cup v_{i}.s_{i}.f_{next,i},\;1<i<\left|\forall w\in f\right|\\ f_{1} & =f_{1}\cup f_{prev,i},\;1<i<\left|\forall w\in f\right|\\ \tau_{2} & =\tau_{2}\cup v_{i}.s_{i}.f,\;1<i<\left|\forall w\in f\right| \end{array}$$
    and return *τ*_*u*_ ← (*τ*_1_, *τ*_2_, *f*_1_, *f*_2_ = *f*)
3. When the update operation is AddKeyword *w* ∈ *W* to document *f* then compute:
  - Get $v\leftarrow_{\$}V_{w},r\leftarrow_{\$}Z_{n},v^{\prime }\leftarrow_{\$}V_{r}$, compute *s* ← Enc_2_(pk, *w*, *r*)
  - When *f* is the header node, compute *τ*_1_ ← *v*.*s*.*f*, *τ*_2_ ← *v*.*s*.*f*_*next*_ and return *τ* ← (*τ*_1_, *τ*_2_, *f*_1_ = 0, *f*_2_ = *f*)
  - When *f* is the terminal node, compute *τ*_1_ ← *v*.*s*.*f*, *τ*_2_ ← *v*.*s*.⊥ and return *τ* ← (*τ*_1_, *τ*_2_, *f*_1_ = *f*_*prev*_, *f*_2_ = *f*)
  - When *f* is an intermediate node, determine *f*_*next*_, *f*_*prev*_ in respect of *f*, compute *τ*_1_ ← *v*.*s*.*f*, *τ*_2_ ← *v*.*s*.*f*_*next*_ and return *τ*_*u*_ ← (*τ*_1_, *τ*_2_, *f*_1_ = *f*_*prev*_, *f*_2_ = *f*)
4. When the update operation is AddFile for a document *f* then compute:
  - Get $v\leftarrow_{\$}V_{w},r\leftarrow_{\$}Z_{n},v^{\prime }\leftarrow_{\$}V_{r}$
  - Compute *s* ← Enc_2_(pk, *w*, *r*)
  - Add *f* as the header node by computing
    $$\begin{array}{cc} \tau_{1}=\sum_{i=1}^{\left|w\in f\right|}v_{i}.s_{i}.f, & \;\tau_{2}=\sum_{i=1}^{\left|w\in f\right|}v_{i}.s_{i}.f_{next,i} \end{array}$$
    and return *τ*_*u*_ = (*τ*_1_, *τ*_2_, *f*_1_ = 0, *f*_2_ = *f*)

### 3.9 Algorithm *γ*′ ← Update(*γ, τ_u_*, *f*_1_, *f*_2_)

This algorithm takes an index *γ*, an update token *τ*_*u*_ = (*τ*_1_, *τ*_2_) and the public key pk as input and updates the encrypted index. The updated index *γ*′ is computed by Update algorithm is given below:

1. When the update operation *u* ∈ {DeleteKeyword, DeleteFile}
  $$\begin{array}{c} \gamma^{\prime }=\{\begin{array}{cc} \gamma \left(f\right)=\gamma \left(f\right)+k-\tau_{2}, & \forall k\in \tau_{1},\forall f\in f_{1}\\ \gamma \left(f_{2}\right)=\gamma \left(f_{2}\right)-\tau_{1}, & \forall k\in \tau_{1} \end{array} \end{array}$$
2. When the update operation *u* ∈ {AddKeyword, AddFile}
  $$\begin{array}{c} \gamma^{\prime }=\{\begin{array}{c} \gamma \left(f_{1}\right)=\gamma \left(f_{1}\right)+\tau_{1}-\tau_{2}\\ \gamma \left(f_{2}\right)=\gamma \left(f_{2}\right)+\tau_{2} \end{array} \end{array}$$

**Fig 4:**
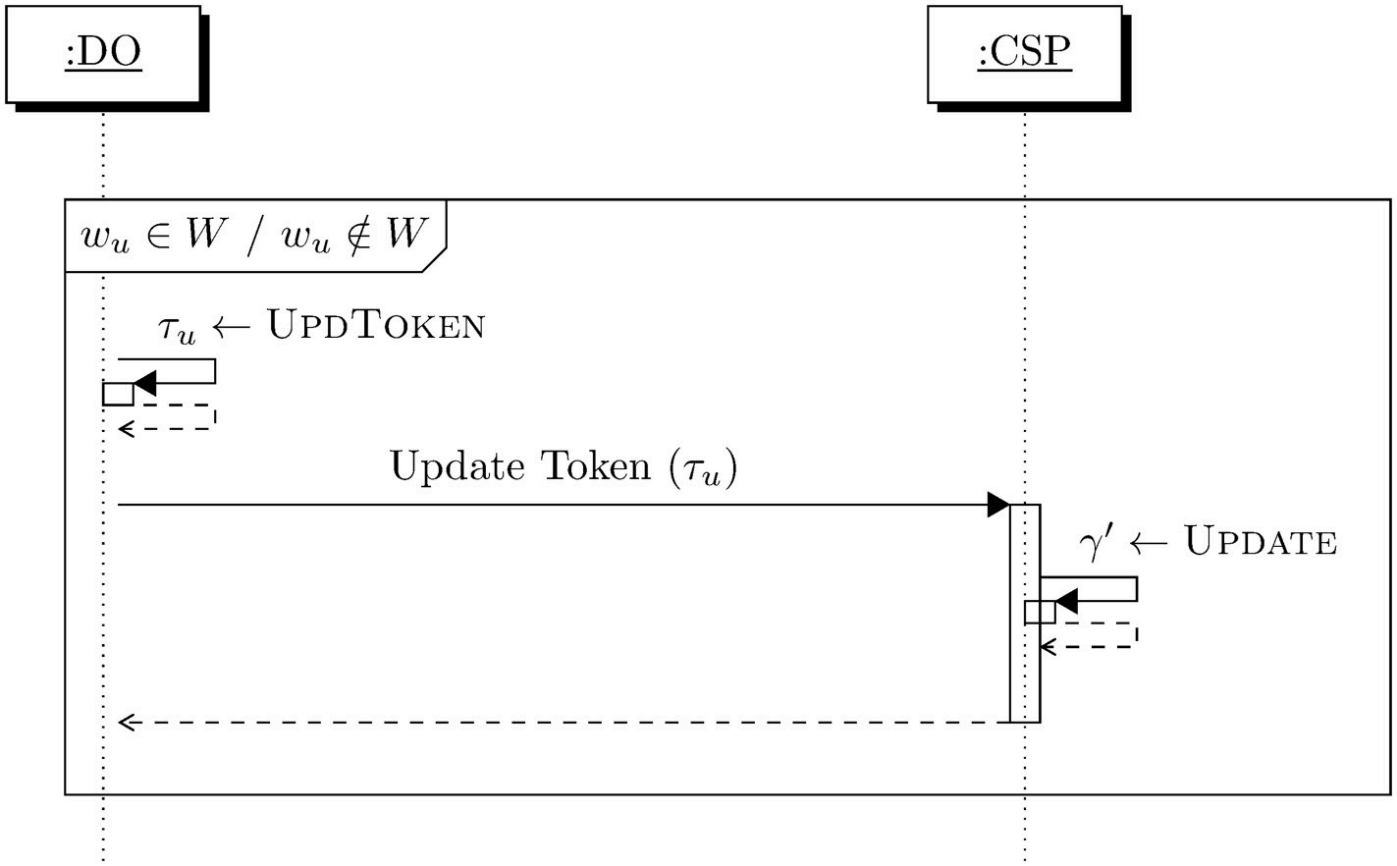
Update working.

The rationale behind the design of Update described above and depicted in Fig 4 is based on exploiting the orthogonal property of *V* and homomorphic property of *s* used in creating the index (*γ*, Γ). This enables addition and deletion over encrypted index such that the tokens are indistinguishable guaranteed by CPA-security.

## 4 Security analysis

The privacy guarantees and correctness of the proposed design, Pindex with respect to the security model given in section 2 and particularly in definition 4 is analysed in this section. The first formal security model given by Bellare et al. [19] is a game-based definition in which the adversary is allowed to interact with a set of oracles (encryption and decryption). The definition models communicating parties in a network where the adversary’s goal is to distinguish between a correct shared secret or random value for the given challenge. Similar approach is used to prove the indistinguishability of the allowed information leakage of the protocol Pindex. Firstly, the correctness of the proposed protocol is proved independently. Secondly, the privacy and correctness with respect to the security model is proved. The Search can handle only two cases: *w*_*q*_ ∈ *W* and *w*_*q*_ ∉ *W* during its operation. To prove the correctness in each case, the proof follows two lemmas:

**Lemma 1**. *When given a search token*$\tau_{w_{q}}$*for a keyword* (*w*_*q*_ ∉ *W*) *and the index γ, the* $\text{Search}(\gamma ,\tau_{w_{q}})$ *returns* ⊥ *such that*

$$\begin{array}{c} \text{Pr}[F_{q}\leftarrow \text{Search}\left(\gamma ,\tau_{w_{q}\notin W}\right):F_{q}={\left\{⊥\right\}}_{w_{q}\notin W}]=1 \end{array}$$

*Proof*. The concise representation of the Search algorithm for the above case is given below:

$$\begin{array}{cc} f_{l} & =\gamma \left(0\right).\tau_{w_{q}}=\left(\sum_{i=1}^{\left|W\right|}v_{i}.s_{i}.f_{z_{i}}+v_{r}.r\right).\left(v_{q}.s_{q}^{-1}+v_{r}.r\right)\\ & =\left(\sum_{i=1}^{\left|W\right|}v_{i}.{\text{Enc}}_{2}\left(\text{pk},w_{i},r_{i}\right).f_{z_{i}}+v_{r}.r\right).\left(v_{q}.{\text{Enc}}_{2}\left(\text{pk},-w_{q},r_{q}^{-1}\right)+v_{r}.r\right)\\ & =v_{i}.v_{q}.{\text{Enc}}_{2}\left(\text{pk},w_{i},r_{i}\right).{\text{Enc}}_{2}\left(\text{pk},-w_{q},r_{q}^{-1}\right).f_{z_{i}}=0 \end{array}$$

When *i* ≠ *q*, *v*_*i*_.*v*_*q*_ = 0 and ∀(*v*_*i*_, *v*_*q*_) ∉ *V*, *v*_*i*_.*v*_*q*_ = 0. It can be observed from the Search algorithm that ⊥ is returned whenever *f*_*l*_ = 0. Therefore, whenever a query keyword *w*_*q*_ ∉ *W* is searched over the document set the Search algorithm returns ⊥ with probability 1.

**Lemma 2**. *When given a search token*$\tau_{w_{q}}$*for a keyword* (*w*_*q*_ ∈ *W*) *and the index γ, the* $\text{Search}(\gamma ,\tau_{w_{q}})$ *returns* $F_{q}={\left\{f_{i}\right\}}_{w_{q}\in W}^{i=1,..|w_{q}\in W|}$ *such that*

$$\begin{array}{c} \text{Pr}[F_{q}\leftarrow \text{Search}\left(\gamma ,\tau_{w_{q}\in W}\right):F_{q}={\left\{f_{i}\right\}}_{w_{q}\in W}^{i=1,..|w_{q}\in W|}]=1 \end{array}$$

*Proof*. Similar to lemma 1, the Search algorithm can be generally represented for *w*_*q*_ ∈ *W* as:

$$\begin{array}{cc} f_{l} & =\gamma \left(0\right).\tau_{w_{q}}=\left(\sum_{i=1}^{\left|W\right|}v_{i}.s_{i}.f_{z_{i}}+v_{r}.r\right).\left(v_{q}.s_{q}^{-1}+v_{r}.r\right)\\ & =(\left(\sum_{i=1}^{\left|W\right|}v_{i}.{\text{Enc}}_{2}\left(\text{pk},w_{i},r_{i}\right).f_{z_{i}}+v_{r}.r\right).\left(v_{q}.{\text{Enc}}_{2}\left(\text{pk},-w_{q},r_{q}^{-1}\right)+v_{r}.r\right))\\ & =v_{i}.v_{q}.g^{w_{i}}.r_{i}^{n}.g^{-w_{q}}.{\left(r_{q}^{n}\right)}^{-1}.f_{i}\;\mathrm{mod}\;n^{2}=f_{i} \end{array}$$

When *i* = *q*, *v*_*i*_.*v*_*q*_ = 1 for all (*v*_*i*_, *v*_*q*_)∈*V* and $g^{w_{i}-w_{q}}=g^{0}=1$ and $r_{i}^{n}.{\left(r_{q}^{n}\right)}^{-1}=1$. Thus, a file identifier *f*_*i*_ will be obtained by the Search algorithm. Therefore, when the computation is recursively repeated until *f*_*l*_ == ⊥, the algorithm returns the set of file identifiers $\left(F_{q}={\left\{f_{i}\right\}}_{w_{q}\in W}^{i=1,..|w_{q}\in W|}=\{f_{1},f_{n}\right\}$ with probability 1.

**Lemma 3**. *The algorithm* Update *provides correct dynamic index update for a given update token τ*_*u*_.

*Proof*. The Update algorithm updates the encrypted index using an update token generated for both the cases addition or deletion of files/keywords.

Case DeleteKeyword: The process of deleting a keyword is as follows:

1. When *f* is the header node file pointer, the UpdateToken computes *τ*_1_ ← *v*.*s*.*f*_*next*_, *τ*_2_ ← *v*.*s*.*f* and returns *τ*_*u*_ ← (*τ*_1_, *τ*_2_, *f*_1_ = 0, *f*_2_ = ⊥). The Update is represented as:
  $$\begin{array}{c} \gamma (f_{1})=\gamma \left(f_{1}\right)+\tau_{1}-\tau_{2}=\gamma \left(0\right)+v_{w_{u}}.s_{w_{u}}.f_{next}-v_{w_{u}}.s_{w_{u}}.f \end{array}$$ (10)
2. When *f* is the terminal node, compute *τ*_1_ = *v*.*s*.⊥, *τ*_2_ = *v*.*s*.*f* and return $\tau_{u}=\left(\tau_{1}={\left\{k\right\}}_{i=1}^{\left|w\in f\right|},\tau_{2},f_{1}={\left\{f_{prev,i}\right\}}_{i=1}^{\left|w\in f\right|},f_{2}=⊥\right)$
  $$\begin{array}{c} \gamma (f_{1})=\gamma \left(f_{1}\right)+\tau_{1}-\tau_{2}=\gamma \left(f_{prev}\right)+v_{w_{u}}.s_{w_{u}}.⊥-v_{w_{u}}.s_{w_{u}}.f \end{array}$$ (11)
3. When *f* is a neither a header nor a terminal node pointer, determine *f*_*next*_, *f*_*prev*_, compute *τ*_1_ ← *v*.*s*.*f*_*next*_, *τ*_2_ ← *v*.*s*.*f* and return $\tau_{u}\leftarrow \left(\tau_{1}={\left\{k\right\}}_{i=1}^{\left|w\in f\right|},\tau_{2},f_{1}={\left\{f_{prev,i}\right\}}_{i=1}^{\left|w\in f\right|},f_{2}=⊥\right)$
  $$\begin{array}{c} \gamma \left(f_{1}\right)=\gamma \left(f_{1}\right)+\tau_{1}-\tau_{2}=\gamma \left(f_{prev}\right)+v_{w_{u}}.s_{w_{u}}.f_{next}-v_{w_{u}}.s_{w_{u}}.f \end{array}$$ (12)

Eqs 10, 11 and 12 demonstrates how the file pointers are adjusted when the keyword *w*_*u*_ has to be added or removed. The old pointers are removed and new pointers are updated from/to previous or next appropriate file depending on the case.

Case AddKeyword: The process of adding a keyword can be represented as follows:

1. When *f* is the first node, compute *τ*_1_ ← *v*.*s*.*f*, *τ*_2_ ← *v*.*s*.*f*_*next*_ and return *τ*_*u*_ ← (*τ*_1_, *τ*_2_, *f*_1_ = 0, *f*_2_ = *f*)
  $$\begin{array}{cc} \gamma (f_{1}) & =\gamma \left(f_{1}\right)+\tau_{1}-\tau_{2}=\gamma \left(0\right)+v_{w_{u}}.s_{w_{u}}.f_{next}-v_{w_{u}}.s_{w_{u}}.f\\ \gamma (f_{2}) & =\gamma \left(f_{2}\right)+\tau_{2}=\gamma \left(f\right)+v.s.f_{next} \end{array}$$ (13)
2. When *f* is the terminal node, compute *τ*_1_ ← *v*.*s*.*f*, *τ*_2_ ← *v*.*s*.⊥ and return *τ*_*u*_ ← (*τ*_1_, *τ*_2_, *f*_1_ = *f*_*prev*_, *f*_2_ = ⊥)
  $$\begin{array}{c} \gamma (f_{1})=\gamma \left(f_{1}\right)+\tau_{1}-\tau_{2}=\gamma \left(f_{prev}\right)+v_{w_{u}}.s_{w_{u}}.f-v_{w_{u}}.s_{w_{u}}.⊥ \end{array}$$ (14)
3. When *f* is a neither a header nor a terminal node pointer, determine *f*_*next*_, *f*_*prev*_, compute *τ*_1_ ← *v*.*s*.*f*, *τ*_2_ ← *v*.*s*.*f*_*next*_ and return *τ*_*u*_ ← (*τ*_1_, *τ*_2_, *f*_1_ = *f*_*prev*_, *f*_2_ = *f*)
  $$\begin{array}{cc} \gamma (f_{1}) & =\gamma \left(f_{1}\right)+\tau_{1}-\tau_{2}=\gamma \left(f_{prev}\right)+v_{w_{u}}.s_{w_{u}}.f-v_{w_{u}}.s_{w_{u}}.f_{next}\\ \gamma (f_{2}) & =\gamma \left(f_{2}\right)+\tau_{2}=\gamma \left(f\right)+v.s.f_{next} \end{array}$$ (15)

Eqs 13, 14 and 15 shows how the file pointers are modified while adding the keyword *w*_*u*_. File addition AddFile and deletion DeleteFile follow similar modification of file pointers. Hence, the dynamic index update is performed correctly.

To prove the indistinguishability between the real game and the ideal game by any *PPT* distinguisher *Dist*, we construct a *PPT* simulator Sim which uses the leakage functions $L_{1}$ and $L_{2}$ to simulate functionality correctly of the algorithms described in Definition 1 indistinguishablly.

**Theorem 1**. *Given the Definition(CKA-2 security) 4, the* Pindex *scheme given above is* $(L_{1},L_{2})-$ *secure in the random oracle model, where* $L_{1}$ *leaks the number of keywords, the number of documents, the identifiers of the documents and the size of each document; and* $L_{2}$ *leaks the search pattern and the access pattern*.

*Proof*. Let Sim be the simulator which interacts with an adversary A in an execution of an ${\text{Ideal}}_{A,\text{Sim}}\left(k\right)$ as in Definition 4. We construct (*γ*, *c*) given the leakage $L_{1}\left(\delta ,f\right)$ as follows:

1. Given the number of files *n* and size of each file *l* through the leakage function, the Sim obtains *c* = (*c*_1_,…, *c*_*n*_) by simulating the encryption of files using ${\text{Sim}}_{{\text{Enc}}_{1}}$ which exists by definition of CPA-Secure Enc_1_.
2. Given the file identifiers *F* = (*f*_1_,…, *f*_*n*_) through the leakage function, the Sim simulates the index *γ*:
  - The Sim builds the multi-linked list *δ* by generating a order *m* hadamard matrix, *V* where *m* is the number of keywords in the universal keyword set. If no such order *m* exists then the least greatest order to *m* is chosen.
  - Construct (*n* + 1) × *m* state matrix *S* where each entry *S*_*ij*_ ∈ {0,1} is determined by flipping a coin.
  - Compute $\gamma \left(0\right)\leftarrow \sum_{j=1}^{m}v_{j}.{\text{Enc}}_{2}\left(\text{pk},j,r\right).f_{x}$ such that *S*_*ij*_ ≠ 0 and *x* = sup(*X*) where $X=\left\{x:S_{yj}\neq 0\wedge 1<y\leq \left(n+1\right)\right\},v_{j}\leftarrow_{\$}V_{w},v_{r}\leftarrow_{\$}V_{r}$ and *r* is obtained from random oracle *H* given *j*. Store *δ*(*j*) ← (*v*_*j*_, *r*).
  - Compute $\gamma \left(f_{i}\right)\leftarrow \sum_{j=1}^{m}v_{j}.{\text{Enc}}_{2}\left(\text{pk},j,r\right).f_{x}$ for all 1 ≤ *i* ≤ *n* + 1 such that *S*_*ij*_ ≠ 0 and if sup(*X*) ≠ ∅ then *x* = sup(*X*) otherwise *x* = ⊥ where $X=\left\{x:S_{yj}\neq 0\wedge i<y\leq \left(n+1\right)\right\},\left(v_{j},r\right)\leftarrow \delta \left(j\right)$.
3. Sim then gives adversary A the encrypted index *γ*.
4. When the Sim receives an adaptive query *q* for keyword *w*_*j*_, it computes *τ*_*q*_ ← *v*_*j*_.Enc_2_(pk, −*j*, *r*^−1^) + *v*_*r*_.*r*′ and returns *τ*_*q*_ to the adversary A. The A may use the Search function for the obtained token *τ*_*q*_ and it will obtain exactly the same file identifiers as in the leakage function $L_{2}$ every time correctly although the search token are different for same keyword *w*_*j*_. It repeats only after making |*V*_*r*_| queries for the same keyword *w*_*j*_.
5. When the Sim receives an adaptive update query *q* = *u* = *f*_*i*_ and if *u* is for add, the simulator uses ${\text{Sim}}_{{\text{Enc}}_{1}}$ to simulate *c*_*i*_ assuming that the leakage function $L_{1}$ reveals the identifier and performs:
  - Updates the state by adding a row to the state matrix *S* where *q* = *n* + 2 and each entry *S*_*qj*_ ∈ {0,1} is determined by flipping a coin.
  - When *f* is first row and *j* is the keyword, compute *τ*_1_ ← *v*.Enc_2_(pk, *j*, *r*).*f*, *τ*_2_ ← *v*.Enc_2_(pk, *j*, *r*).*f*_*next*_ where *next* = sup(*X*) and *X* = {*next*: *S_yj_* ≠ 0 ∧ 1 < *y* ≤ (*n* + 1) } and (*v*, *r*) ← *δ*(*j*). Returns *τ* ← (*τ*_1_, *τ*_2_, *f*_1_ = 0, *f*_2_ = *f*).
  - When *f* is the terminal row and *j* is the keyword, compute *τ*_1_ ← *v*.Enc_2_(pk, *j*, *r*).*f*, *τ*_2_ ← *v*.Enc_2_(pk, *j*, *r*).⊥. Returns *τ* ← (*τ*_1_, *τ*_2_, *f*_1_ = *f*_*prev*_, *f*_2_ = ⊥) where *prev* = inf(*X*) and *X* = {*prev*: *S*_*yj*_ ≠ 0 ∧ *i* > *y* > 0} and (*v*, *r*) ← *δ*(*j*).
  - When *f* is a neither a header nor a terminal node pointer, determine *f*_*next*_, *f*_*prev*_, compute *τ*_1_ ← *v*.*s*.*f*, *τ*_2_ ← *v*.*s*.*f*_*next*_ where *prev* = inf(*X*) and *X* = {*prev*: *S*_*yj*_ ≠ 0 ∧ *i* > *y* > 0} while *next* = sup(*X*) and *X* = {*next*: *S_yj_* ≠ 0 ∧ *i* < *y* ≤(*n* + 1)} and (*v*, *r*) ← *δ*(*j*). Returns *τ*_*u*_ ← (*τ*_1_, *τ*_2_, *f*_1_ = *f*_*prev*_, *f*_2_ = *f*).
  - A can then use *τ*_*u*_ on Update function and obtain the updated encrypted index *γ*′.
6. Similarly, Sim can simulate operation Add and Delete for keywords and file.

The results returned to the A’s random oracle queries by Sim are consistent. The keys used in the tokens and keys used in the construction of index *γ* are indistinguishable since (Enc_1_,Enc_2_) used to encrypt is by definition indistinguishable from random. Hence, the CPA-Security of (Enc_1_,Enc_2_) guarantee indistinguishability between real and simulated encryptions of the files and index by A.

## 5 Performance analysis

**Fig 5:**
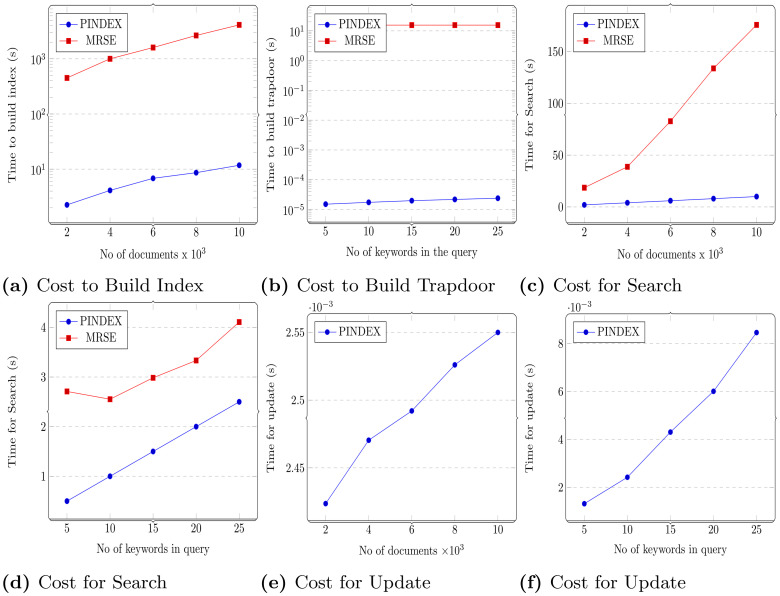
Experimental results.

This section presents the performance analysis of the proposed design for its efficiency. Pindex is implemented in Java and the experimental results are evaluated on a Windows server with Intel Xeon Processor running at 2.30 GHz and 16 GB RAM. The experiment uses real world Enron email dataset [20]. The performance of Pindex is evaluated and compared with MRSE [21]. The experimental results are shown in Fig 5.

### 5.1 Multi-link index

Building the multi-linked index is a one-time process carried out by the data owner. The multi-linked dynamic searchable index *γ* can be constructed using two steps. First, encrypting the keyword *w* using Paillier cryptosystem if it belongs to document *f* and secondly computing the encrypted index *γ* which is a multi-link between the document *f* and keywords *w*_*i*_ ∈ *W*. Therefore the time cost of index generation is proportional to the number of files and number of keywords contained in each file. It can be observed from Fig 5a that the time cost for building the index with varying number of documents of the proposed system performs relatively better than MRSE. This is due to use of relatively small matrix dimensions in the design and computationally less intensive operations like scalar multiplications (|*W*| operations) and vector additions (|*W*| + 1 operations).

### 5.2 Search token generation

The search token *τ*_*q*_ is constructed for the keywords in the query. The process involves retrieving the pair (*v*, *r*) from the parameter hash table Γ and encrypting the query keyword *w*_*q*_. The time cost for search token depends on the number of query keywords. Fig 5b shows the time cost for generating the search token in logarithmic scale for document size *n* = |*D*| = 1000 and varying the size of the query keyword. It is observed that Pindex performance is better than MRSE. This is because our scheme use less computationally intensive operations and the number of operations involved in building a trapdoor is minimum. The operations include two scalar multiplications, one vector addition for indistinguishability and one lookup.

### 5.3 Search

Search operation executed by the cloud server consists of multiplying the search token *τ*_*q*_ with the encrypted index *γ*. If *w*_*q*_ ∈ *W*, the first pointer is computed and recursively the multi linked index is traversed until *f*_*q*_ = ⊥. For a multi-keyword search with |*w*_*q*_| keywords in a query *q* and run on *p* processors parallel which returned |*F*_*q*_| files then time complexity would be $O(|F_{q}|/p)$. In MRSE similarity scores for the document set is computed and therefore the time complexity is O(N). The time cost for varying the number of documents for *m* = |*W*| = 1000 is shown in Fig 5c and 5d. Search time of Pindex is relatively better than MRSE. Also, it can be observed that as the number of keywords per query is increased, the corresponding increase in time of query to be sub-linear for the proposed design. It is due to optimal number of operations used in the design of search operation which involves just one vector inner product.

### 5.4 Update

The update (add and delete) of both keywords and document involves adding the update token *τ*_1_ and deleting *τ*_2_ from *f*_*prev*_. The worst case scenario for update delete file operation is when the number of *f*_*prev*_ equals the number of keywords contained in the file to be deleted. Updates are not compared as MRSE does not support dynamic index, the index has to be rebuilt for updates and the results are shown in Fig 5e and 5f. The time cost for update i.e to add/ delete a keyword is O(1) and to add/delete a file is O(k/p). The proposed method supports dynamic updates unlike MRSE and it can be observed that dynamic updates comprises of building update trapdoor and executing update algorithm. The time to build update trapdoor is significantly less due to the same reasoning as that of building trapdoor and execution of update algorithm involves only vector additions and deletions as given in section 3.

## 6 Related works

The confidentiality of sensitive data like Electronic Health Records (EHR) outsourced to CSP is protected from external and internal attacks by deploying a searchable encryption scheme specially designed to support operations like search and updates over encrypted data [22].

### 6.1 Searchable encryption

The searchable encryption schemes are primarily classified into Searchable Symmetric Encryption and Public Key Encryption with Keyword Search [2] depending upon the type of encryption primitive used and notion of provable security. The searchable encryption systems is further classified based on the type of search operation into keyword based and semantic based searchable encryption system [23]. The keyword based searchable encryption system uses either specially designed secure encrypted indexes to support search operations over encrypted data or sequential scanning of encrypted documents to support search [4]. The keyword based searchable encryption system are exact search keyword based while the semantic keyword search matches even closest query keywords. The proposed design is based on keyword based searchable encryption schemes using secure encrypted index. The first practical solution to symmetric searchable encryption was proposed by Song et al. [3]. Followed by a number of searchable encryption schemes especially with secure index construction, improved efficiency, security definitions and formalizations [4, 5, 8, 9, 24–26].

### 6.2 Secure index

To improve performance and decrease the search complexity, clients build keyword-based indexes and outsource it to the cloud. Searchable encryption schemes in literature use a forward, inverted or tree-based index [4, 24, 27, 28]. However, the scalability of the index based schemes can be achieved by either rebuilding the index or by using expensive techniques [5]. The index contains information such as document identifiers, document length, number of keywords in the file. The adversary should not be able to determine the query keyword using statistical analysis. Therefore, the index must not leak information which could aide honest-but-curious CSP to link tokens, keywords and document identifiers. The indexes and the tokens or trapdoors are inherently linked. Deterministic trapdoors are known to leak information therefore probabilistic trapdoors are used. Goh [4] uses forward index using bloom filters per document. The search complexity is proportional to the number of files and bloom filters have an inherent problem of false positives. Chang and Mitzenmacher [24] use prebuilt dictionary of distinct keywords to build the forward index. The search complexity is proportional to the number of files. Inverted index or index per keyword was introduced by [5] and thus reduced the search time to a sub-linear scheme with optimal search time. [26] proposed a scheme based on inverted index using hash tables.

### 6.3 Dynamic searchable encryption

Dynamism is a main requirement for any searchable encryption scheme to be practical. The first dynamic searchable encryption was proposed by Kamara et al. [8] using inverted index and dynamic addition and deletion of documents. The update complexity is linear to the updated document/keyword pair but the update leaks some information about the documents. Kamara and Pamamanthou [9] proposed a red-black tree index based on an encrypted linked-list multi-map. To insert a new keyword, they added as a new entry to the right list and deletion using a dual representation of the index but update leaks information. Cash et al. [10] proposed a dynamic index using a separate update database to add and delete tuples stored in the revocation list. However, the update is inefficient and leaks significant information. Naveed et al. [11] presented a dynamic searchable encryption with blind storage. Instead of encrypting the index, the stored blocks are scattered using hashing. This scheme leaks the information while adding keyword. Other dynamic searchable encryption schemes are [7, 14, 29–31].

### 6.4 Forward privacy

There is a trade-off between practicality and security as most of the dynamic searchable encryption schemes leak significant amount of information about the document. Attacker can leverage this leakage to reveal information on the client’s queries. Deterministic encryption schemes leak information regarding size, search, access patterns from the repetition of the queried keyword. File injection attack on dynamic searchable encryption by Zhang et al. [13] triggered much attention on forward privacy schemes. By injecting few carefully selected files, the adversary can recover keywords queried in the past from the information leaked from the search token. Thus, the prior search token needs to be invalidated to achieve forward security. Therefore, a stronger property called forward privacy and an informal definition was introduced by Stefanov et al. [12] based on Oblivious RAM. However, the scheme leads to a search cost $O(|F_{q}|\log^{3}N)$ and $O(k\log^{2}N)$ update cost. Existing schemes that achieve forward privacy are generally based on trapdoor permutation or using pseudorandom functions. Bost proposed the first formal definition of forward security and a trapdoor permutation based scheme *Sophos* [32]. Later proposed *Diana* [15] a constrained PRF based scheme. A dual dictionary *Dual* [33] was proposed that simultaneously uses both forward with inverted index and achieves forward privacy using fresh keys. To realize forward security, a symmetric punctured encryption *Janus ++* [34] was proposed. Etemad et al. [35] achieve forward privacy by replacing the keys revealed to the server. Sun et al. *Aura* [36] use a non-interative DSSE using bloom filters and multi-puncturable PRF. Wei et al. [37] proposed an index structure with keyed block chain. *Khons* [38] uses inverted index and achieves weak forward security by exploitation hidden pointer technique.

## 7 Discussion

A comparison of our work with existing dynamic schemes is given in Table 1. In our work, there is a relative improvement in client and server storage. The client storage is O(n) as the client needs to store the *δ*, which is the sum of the inner product of orthogonal vectors for documents and Γ the keyword parameter hash table. The server storage is O(n) as the server stores the encrypted multi-linked index *γ* that contains the number of document and the keywords *w*_*i*_ ∈ *W*. Efficient updates are performed without rebuilding the index. The search and update tokens are indistinguishable from the previous queries. The proposed multi-linked index is parallelizable and provides relatively efficient search and update with $O(|F_{q}|/p)$ and O(k/p) respectively. The efficient time complexity of search is achieved by exploiting the vertical pointers and orthogonal horizontal linking property of the proposed multi-linked encrypted index structure which enables retrieval of the encrypted keyword index with a vector inner product. It can be observed from the SrchToken algorithm that for each search, the token generated consists of the orthogonal sum of $\left(v_{i}.s_{i}^{-1}\right)$ and (*v*_*r*_.*r*) in which (*v*_*r*_, *r*) is chosen uniformly random for each query to make the token indistinguishable for even the same query. Similarly, the UpdToken algorithm also generates each token which are indistinguishable. This token generation strategy makes the tokens, file identifiers and keywords unlinkable which eventually provides forward privacy as in Definition 5. Moreover, the search can be performed in parallel for multiple tokens simultaneously in parallel on a shared index. This makes the proposed scheme more efficient in exploiting the available parallel processors and improves the execution speed of search operations.

**Table 1:** Complexity and feature comparison.

Scheme	Client storage	Server storage	Search Cost	Update Cost	Forward	Parallel
**Kamara et al**. [8]	O(1)	O(N)	$O(|F_{q}|)$	O(k)	No	No
**Parallel SSE** [9]	O(1)	O(mn)	$O(|F_{q}|\log n)/p)$	O(mlogn/p)	No	Yes
**Blind Storage** [11]	O(1)	O(N)	$O(|F_{q}|+n_{d})/p)$	O(k/p)	No	Yes
**Cash et al**. [10]	O(m)	O(N)	$O(|F_{q}|+n_{ad})/p)$	O(k/p)	No	Yes
**Practical SSE** [12]	$O(\sqrt{N})$	O(N)	$O(|F_{q}|\log^{3}N)$	$O(k\log^{2}N)$	Yes	No
**Sophos** [32]	O(m)	O(N)	$O(|F_{q}|+n_{ad})$	O(k)	Yes	No
**Etemad et al**. [35]	O(m+n)	O(N)	$O(|F_{q}|+n_{d})/p)$	O(k/p)	Yes	Yes
**Ours**	O(n)	O(n)	$O(|F_{q}|/p)$	O(1)/O(k/p)	Yes	Yes

*n*—total number of files, *m*- total number of keywords, |*F*_*q*_|—number of files containing a multiple keywords, *k*—number of unique keywords in a file. *p* and *N* are number of processors and (keyword, file ID) mappings respectively, *n*_*ad*_ and *n*_*d*_—number of times a keyword has been affected by file deletions since beginning and since the last search for the same keyword, respectively and (*n*_*ad*_ ≥ *n*_*d*_).

## 8 Conclusion

This paper proposes a novel private multi-linked dynamic index construction for efficient retrieval of encrypted documents with forward privacy guarantees. The multi-linked index is constructed using probabilistic homomorphic encryption and secret orthogonal vectors. Experimental evaluations on Enron dataset proves that our construction achieves optimal search and update cost. Pindex supports parallelism using the multi-link structure. Thus, the server can distribute the load to its available *p* processors with search and update cost as $O(|F_{q}|/p)$ and O(k/p) respectively. Forward privacy guarantees is achieved using probabilistic algorithms for search and update token generation. Further, the client and server storage is reduced to O(n). Security analysis of Pindex using random oracle models proves the privacy and correctness of search and update operations. Our work Pindex can be used on critical cloud infrastructre for secure and efficient encrypted document retrieval with forward privacy guarantees. As a future work, Pindex can be adapted to provide verifiablilty and backward privacy.
